# Microglial PD-1/PD-L1 axis in CNS demyelinating diseases: a dual immunoregulatory perspective

**DOI:** 10.3389/fncel.2026.1815351

**Published:** 2026-04-08

**Authors:** Meiling Zhou, Xiying Yao, Lingchun Liu, Yuan Gao, Wenli Chen, Kunwen Zheng, Haixia Li, Qiang Meng

**Affiliations:** 1 Department of Neurology, The Affiliated Hospital of Kunming University of Science and Technology, The First People's Hospital of Yunnan Province, Kunming, Yunnan, China; 2 School of Medicine, Kunming University of Science and Technology, Kunming, China

**Keywords:** immunotherapy, microglia, multiple sclerosis, neuromyelitis optica spectrum disorder, PD-1/PD-L1 axis

## Abstract

Multiple sclerosis (MS) and neuromyelitis optica spectrum disorder (NMOSD) stand as archetypal autoimmune-mediated demyelinating diseases of the central nervous system (CNS). Emerging evidence highlights the dual immunomodulatory functions of microglia in these diseases: on the one hand, they can secrete neurotoxic molecules that exacerbate neural damage; on the other hand, they are capable of releasing neuroprotective factors that promote tissue repair and enhance neuronal survival. This review dissects the programmed cell death ligand 1 (PD-L1)/programmed cell death protein 1 (PD-1) immune checkpoint axis, expressed on activated microglia, T cells, and other immune cells, as a pivotal rheostat of neuroinflammation. The binding of PD-1 to PD-L1 dampens immune cell activation and proliferation, curtails pro-inflammatory cytokine output, and is instrumental in preserving immune tolerance. In the context of chronic inflammation, persistent PD-1/PD-L1 signaling has been closely associated with the induction of T cell exhaustion than with direct apoptosis, though context-dependent effects on cell survival have been reported in certain experimental paradigms. Both microglia and the PD-1/PD-L1 axis are critically intertwined in the initiation and perpetuation of CNS demyelinating diseases. A more granular comprehension of their interplay will not only illuminate the molecular underpinnings of neuroinflammation and immune regulation in MS and NMOSD but also pave the way for crafting precision immunotherapies aimed at modulating microglial polarization. Here, we systematically review the dual immunomodulatory functions of the microglial PD-1/PD-L1 axis in these diseases and deliberate on the therapeutic prospects of targeting this pathway, thereby furnishing a conceptual framework for novel immune intervention strategies.

## 1 Introduction

Demyelinating diseases of the CNS, chiefly MS and NMOSD, are chronic autoimmune disorders characterized by inflammatory demyelination and cumulative neurological decline. Their clinical and pathological distinctions, both diseases involve complex interactions between infiltrating peripheral immune cells and resident CNS glial cells, among which microglia play a pivotal role (Chan et al., 2025).

As the brain’s innate immune sentinels, microglia are critical arbiters of neuroinflammation (Chauhan and Lokensgard, 2019). Upon sensing inflammatory cues, these cells do not simply toggle between binary states but undergo dynamic phenotypic polarization, exhibiting a spectrum of activation profiles. In active MS lesions, a pro-inflammatory microglia signature often predominates, with cells releasing IL-1β, TNF-α, and IL-6, cytokines that fuel tissue damage and summon peripheral leukocytes (Chan et al., 2025). Yet, even within acute lesions, striking regional heterogeneity exists; transcriptional profiles of microglia at the lesion edge differ markedly from those in the necrotic core (Smolders et al., 2018). During remission, the balance shifts, and microglia adopt a more reparative phenotype, secreting factors like IL-10 and IGF-1 that encourage tissue repair and remyelination (Béland et al., 2020). This spatial and temporal heterogeneity underscores the complexity of microglial responses in CNS demyelinating diseases.

These immunomodulatory functions are themselves tightly governed by immune checkpoint pathways, with the PD-1/PD-L1 axis emerging as a key player (Feghali and Jackson, 2025; Zhang Q. et al., 2025). PD-L1 expressed on activated microglia can engage PD-1 on T cells, transmitting inhibitory signals that quell T cell proliferation, cytokine production, and cytotoxic activity (Chauhan and Lokensgard, 2019; Feghali and Jackson, 2025). This molecular handshake is crucial for maintaining CNS immune tolerance and preventing runaway inflammation (Zhang Q. et al., 2025; Linnerbauer et al., 2023). Accumulating evidence suggests that dysregulation of the PD-1/PD-L1 pathway contributes to the pathogenesis of both MS and NMOSD, marking it as a promising therapeutic target (Feghali and Jackson, 2025; Linnerbauer et al., 2023). In this review, we aim to explore the immunomodulatory functions of microglia in MS and NMOSD, with a particular focus on the PD-1/PD-L1 signaling pathway’s role in restraining neuroinflammation. We further discuss potential therapeutic strategies targeting this axis, highlighting their clinical implications.

## 2 Immunoregulatory role of microglia via the PD-1/PD-L1 axis in multiple sclerosis

### 2.1 Molecular architecture of the PD-1/PD-L1 pathway and its immunosuppressive function

**Figure 1:**
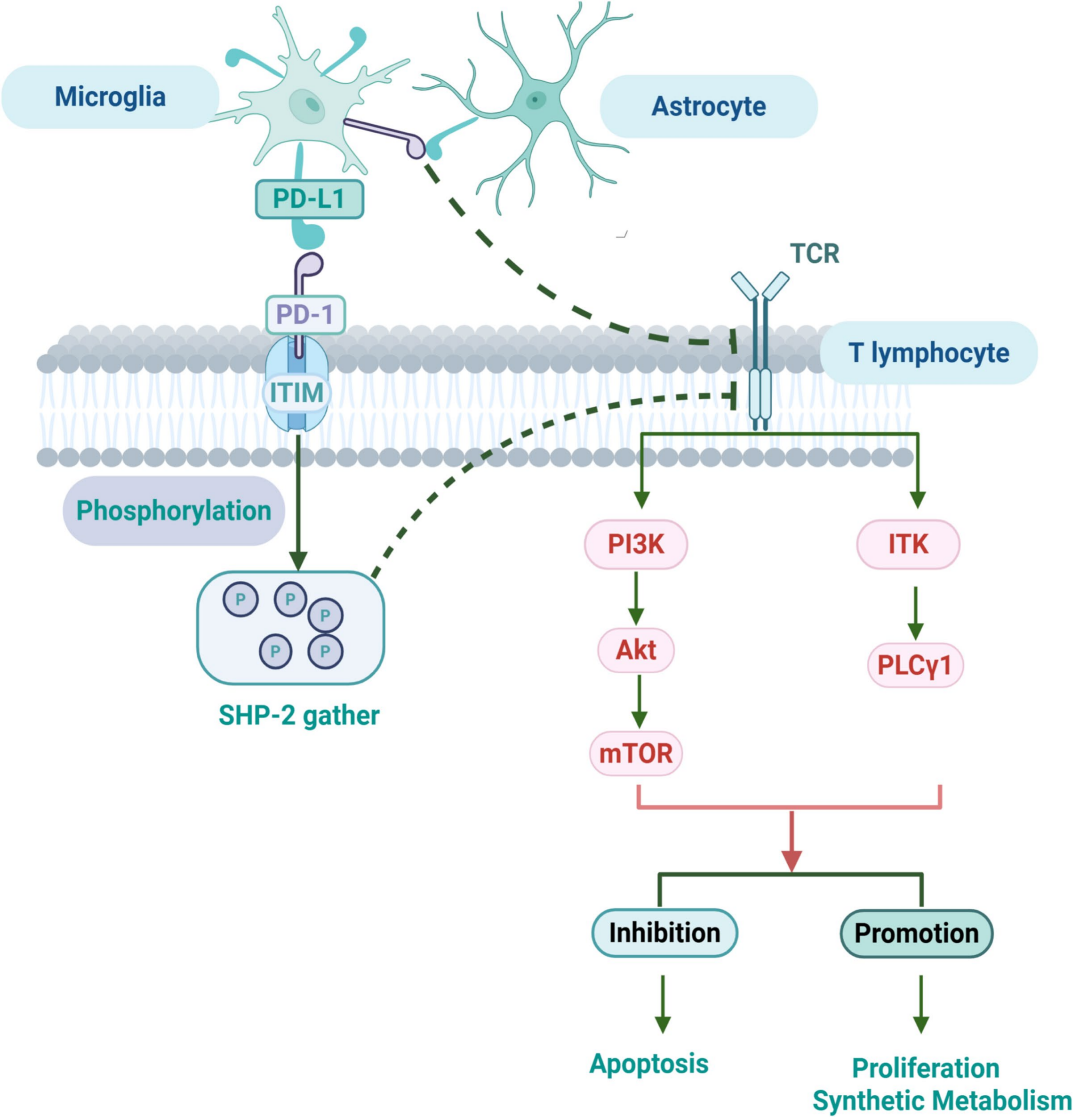
Molecular mechanism of the PD-1/PD-L1 signaling pathway in MS. The binding of PD-1 on the T-cell surface to its ligand PD-L1, expressed on antigen-presenting cells (e.g., microglia), represents a critical immunoregulatory event. This interaction induces phosphorylation of ITIM within the cytoplasmic tail of PD-1, leading to recruitment and activation of the tyrosine phosphatase SHP-2. Activated SHP-2 mediates dephosphorylation of key kinases downstream of the TCR signaling pathway, such as PI3K and ITK, thereby inhibiting T-cell activation, proliferation, cytokine production, and cytotoxic effector functions. Additionally, PD-L1 expressed on astrocytes engages PD-1 on microglia, forming an inhibitory synapse that further restrains inflammatory T-cell responses. Ultimately, activation of the PD-1/PD-L1 pathway suppresses T-cell-mediated immunity, serving as a key mechanism for maintaining immune tolerance and preventing excessive inflammation in the pathogenesis of MS.

PD-1, a prototypical immune checkpoint molecule belonging to the CD28 receptor family, is inducibly expressed on the surface of activated T cells, monocytes/macrophages, B cells, dendritic cells, and NK cells (Zhao et al., 2014). Structurally, this type I transmembrane protein harbors both an immunoreceptor tyrosine-based inhibitory motif (ITIM) and an immunoreceptor tyrosine-based switch motif (ITSM) within its cytoplasmic tail, which orchestrate downstream inhibitory signals upon ligand engagement (Hammond et al., 2019). Its primary ligand, PD-L1 (a member of the B7 family), is similarly distributed on a wide array of immune cells, including T cells, microglia, dendritic cells, and NK cells. The PD-1/PD-L1 axis functions as a critical brake on immune responses. Ligation suppresses the proliferation of inflammatory T cells, inhibits cytokine production, curtails cytotoxic effector functions, and ultimately helps confine CNS inflammation while promoting neural repair (Chauhan and Lokensgard, 2019; Chen et al., 2019). As depicted in Figure 1, this cascade involves recruitment of the phosphatase Src homology 2 domain-containing protein tyrosine phosphatase-2 (SHP-2) to the phosphorylated ITSM/ITIM motifs, leading to dephosphorylation of key signaling nodes downstream of the T cell receptor (TCR), including phosphatidylinositol 3-kinase (PI3K) and interleukin-2-inducible T-cell kinase (ITK), and culminating in inhibition of mechanistic target of rapamycin (mTOR) activation (Mi et al., 2021).

Beyond this canonical scheme, additional layers of regulation fine-tune the system—nuances not captured in Figure 1. First, PD-L1 expression on microglia is subject to post-transcriptional control. A negative feedback loop involving miR-146a has been identified: excessive PD-L1 signaling upregulates this microRNA, which in turn suppresses PD-L1 translation, thereby maintaining physiological levels of this checkpoint molecule (Bhaumik et al., 2009; Yang et al., 2012). This autoregulatory safeguard likely prevents overshoot immunosuppression and helps preserve CNS immune homeostasis.

Second, the PD-1/PD-L1 axis exhibits marked spatiotemporal heterogeneity over the disease course. During acute MS exacerbations, PD-L1 is rapidly upregulated on activated microglia within inflammatory lesions, particularly in perivascular cuffs and active demyelinating plaques (Smolders et al., 2018; Serafini et al., 2024). Conversely, during remission, its expression subsides in tandem with the resolution of inflammation. Spatially, PD-L1 levels also vary across CNS regions; they are more pronounced in areas characterized by dense immune infiltration, such as the spinal cord and optic nerve in NMOSD, compared to relatively immune-privileged zones (Chen et al., 2021; Smith and Williams, 2025). This dynamic expression pattern suggests that the immunomodulatory potency of the PD-1/PD-L1 axis is calibrated according to local microenvironmental cues and disease stage, a nuance with significant implications for therapeutic targeting.

Microglia, which constitute roughly 10% of CNS glial cells, are yolk sac-derived resident macrophages that colonize the brain during embryogenesis. They continuously patrol the CNS parenchyma, playing indispensable roles in maintaining homeostasis, supporting development, and facilitating damage repair (Hammond et al., 2019; Yong, 2022). In the context of MS-related neuroinflammation, microglia rapidly sense inflammatory signals, proliferate, and transition from a quiescent, ramified state to an activated, amoeboid morphology with enhanced phagocytic and migratory capacity (Vermersch et al., 2025; Zrzavy et al., 2017). They upregulate major histocompatibility complex class II (MHC II) and co-stimulatory molecules such as CD80/CD86, effectively functioning as antigen-presenting cells to modulate T lymphocyte responses (Schachtele et al., 2014; Keir et al., 2008). Critically, microglial PD-L1 expression is robustly increased under these conditions. Engagement of PD-1 on infiltrating T cells triggers phosphorylation of the ITIM tyrosine residue within PD-1, recruiting SHP-2 phosphatase and leading to dephosphorylation and inactivation of key TCR-proximal kinases (PI3K, ITK). The consequent inhibition of mTOR activation blocks T cell proliferation and differentiation, suppresses release of inflammatory mediators, and ultimately reins in the inflammatory response (Chauhan and Lokensgard, 2019; Zhao et al., 2014; Lin et al., 2024). Interestingly, astrocytes also contribute to this immunomodulatory network. One study found that during acute MS neuroinflammation, astrocytic PD-L1 expression increases as well. In areas of astrocyte infiltration, PD-1-expressing microglia can form inhibitory synapses with PD-L1^+^ astrocytes, suppressing inflammatory T cells and reducing monocyte infiltration, thereby mitigating CNS inflammation and promoting recovery (Linnerbauer et al., 2023). Functional experiments in MS models have shown that blockade of the PD-1/PD-L1 axis, or suppression of microglial PD-L1 expression, results in enhanced secretion of pro-inflammatory cytokines like IFN-*γ*, IL-2, and IL-17, leading to heightened T cell activation (Ortler et al., 2008; Magnus et al., 2005; Leane et al., 2024), impaired microglial phagocytosis, disrupted differentiation of oligodendrocyte precursor cells, prolonged inflammatory responses, and exacerbated demyelination (Liu et al., 2018).

### 2.2 Dynamic polarization and dual regulatory role of microglia in MS pathogenesis

MS is pathologically defined by white matter inflammation, demyelination, neuronal degeneration, reactive gliosis, blood–brain barrier (BBB) disruption, and axonal injury (Weng et al., 2018). Disease initiation involves activation of the peripheral immune system, where macrophages and dendritic cells present antigens via MHC II to CD4^+^ T cells in lymphoid tissues (Paroni et al., 2017). Activated T cells differentiate into T helper 1 (Th1) and Th17 subsets, releasing large quantities of pro-inflammatory cytokines, unleashing copious amounts of pro-inflammatory cytokines including IFN-γ, TNF-α, and IL-6 (Manel et al., 2008). These cytokines, borne by the circulation, breach the BBB, recruit additional inflammatory cells into the CNS, and amplify neuroinflammation (Yang X. et al., 2021; Raphael et al., 2015).

During active MS, microglia are swiftly activated and show a predominant shift toward a pro-inflammatory, phagocytic state (Pukoli and Vécsei, 2023; Voet et al., 2019; Jack et al., 2005). However, this polarization is far from absolute; single-cell analyses have revealed mixed microglial states even within active plaques, with a continuum of inflammatory and reparative gene expression (Yang et al., 2012; Serafini et al., 2024). At the lesion edge, microglia often exhibit a more activated, phagocytic profile, while those in the core may display signs of cellular stress and senescence (Zrzavy et al., 2017). Upon stimulation by pathogen-associated molecular patterns (PAMPs) or damage-associated molecular patterns (DAMPs), microglia adopt a pro-inflammatory phenotype. This state is characterized by the release of pro-inflammatory mediators (e.g., IL-1, IL-6, TNF, C1q), chemokines (e.g., CCL2, CCL3, CXCL9), and reactive oxygen species (ROS), exacerbating damage to myelin, neurons, and axons, promoting further immune cell infiltration, and worsening MS pathology (Goldmann and Prinz, 2013; Wang et al., 2019; Yang S. et al., 2021).

Beyond their effector functions, pro-inflammatory microglia in the acute phase also serve as antigen-presenting cells, expressing MHC molecules and co-stimulatory markers like CD86, which enhances their ability to activate T cells and perpetuate the immune response (Voet et al., 2019; Jack et al., 2005).

The dynamic balance of microglial polarization is orchestrated by a complex molecular regulatory network involving multiple levels of control. At the transcriptional level, distinct transcription factor cascades drive phenotypic specification. Pro-inflammatory polarization is primarily governed by nuclear factor-κB (NF-κB) and signal transducer and activator of transcription 1 (STAT1), which synergistically promote transcription of pro-inflammatory genes (Shu et al., 2025). Conversely, anti-inflammatory polarization is driven by STAT6 and peroxisome proliferator-activated receptor gamma (PPAR-*γ*), which activate the expression of anti-inflammatory and repair-associated genes (Liu et al., 2024; Yao et al., 2023). The competitive balance between these opposing programs ultimately determines the microglial functional phenotype.

Beyond transcriptional control, metabolic reprogramming has emerged as a fundamental determinant of polarization. Pro-inflammatory microglia undergo a metabolic switch from oxidative phosphorylation to aerobic glycolysis, a phenomenon reminiscent of the Warburg effect in cancer cells, enabling rapid ATP production to support energy-intensive functions like phagocytosis, migration, and cytokine secretion. The mTOR signaling pathway serves as a central hub for this metabolic shift, integrating signals from growth factors, nutrients, and inflammatory cues to orchestrate immunometabolic reprogramming (Chen H. et al., 2025). In contrast, anti-inflammatory microglia primarily rely on oxidative phosphorylation, consistent with their role in tissue repair and homeostasis. This metabolic plasticity is intimately linked to functional phenotype and represents a promising therapeutic target.

Epigenetic mechanisms add yet another layer of regulatory complexity. Histone modifications, particularly acetylation and deacetylation mediated by histone acetyltransferases (HATs) and histone deacetylases (HDACs), dynamically control chromatin accessibility at polarization-related gene loci (Diniz et al., 2025). HDACs have been shown to modulate microglial inflammatory responses by altering histone acetylation at promoter regions of pro-inflammatory genes, and HDAC inhibitors can suppress pro-inflammatory activation while promoting anti-inflammatory phenotypes (Meleady et al., 2023). DNA methylation and non-coding RNAs further fine-tune expression of polarization-associated genes. Notably, miR-155 promotes pro-inflammatory microglia polarization by targeting suppressor of cytokine signaling 1 (SOCS1), while miR-124 facilitates anti-inflammatory polarization by inhibiting C/EBP-*α*, contributing to the stability and reversibility of phenotypic states (Salama et al., 2024; Freilich et al., 2013).

### 2.3 Key molecular switches governing microglial fate: TREM2, and CX3CR1

A growing body of evidence highlights the critical role of membrane-bound receptors that function as “molecular switches” determining microglial activation status and phenotypic direction. These receptors, often conceptualized as immune checkpoints for microglia, mediate continuous crosstalk between microglia and their cellular environment, particularly neurons. Their dysfunction is increasingly recognized as a key driver of neuroinflammation in MS and NMOSD.

Triggering receptor expressed on myeloid cells 2 (TREM2) is an innate immune receptor expressed exclusively on microglia in the CNS. TREM2 binds diverse ligands, including anionic lipids, apolipoproteins, and DAMPs, transmitting signals through its adaptor protein DNAX-activation protein 12 (DAP12) to promote microglial survival, proliferation, and phagocytic activity. In MS, TREM2 signaling is essential for microglial responses to myelin debris and containment of inflammatory damage (Cignarella et al., 2020; Cantoni et al., 2015). Conversely, TREM2 deficiency or dysfunction impairs clearance of myelin debris and exacerbates neuroinflammation. Soluble TREM2 (sTREM2) in cerebrospinal fluid has emerged as a reliable biomarker of microglial activation in both MS and NMOSD (Qin et al., 2024; Kierdorf and Prinz, 2013; Filipello et al., 2022). In NMOSD patients, elevated CSF sTREM2 levels correlate positively with markers of neural injury and disease severity (Qin et al., 2024). Mechanistically, excessive sTREM2-mediated signaling may drive microglial dysfunction through overactivation, overwhelmed phagocytosis, suppressed lipid metabolism, and enhanced glycolysis, possibly via the NF-κB pathway (Zhong et al., 2017). These findings position TREM2 as a critical rheostat: optimal signaling supports protective microglial functions, while its dysregulation, whether through deficiency or excessive shedding, promotes pathogenic states.

The CX3CL1-CX3CR1 axis represents another fundamental neuron–microglia communication pathway (Jung et al., 2000). CX3CL1 (fractalkine) is a transmembrane chemokine constitutively expressed by neurons, while its unique receptor CX3CR1 is exclusively expressed on microglia in the CNS (Cardona et al., 2018; Cardona et al., 2006). Under physiological conditions, this ligand-receptor pair maintains microglia in a quiescent, surveillant state, restraining excessive inflammatory responses. Disruption of CX3CR1 signaling, whether through genetic deficiency or functional impairment, results in microglial dysregulation and exacerbated neurotoxicity (Harrison et al., 1998). In experimental autoimmune encephalomyelitis (EAE), CX3CR1 deficiency confers more severe disease with increased inflammation and neuronal loss (Cardona et al., 2018). Critically, a common human CX3CR1 polymorphism (I249/M280), present in approximately 20% of the population, exhibits reduced adhesion to CX3CL1 and defective signaling (Kezic and McMenamin, 2013). Mice engineered to express this human variant recapitulate the exacerbated EAE phenotype, underscoring the clinical relevance of this pathway (Mai et al., 2019). During neuroinflammation, impaired CX3CR1 signaling leads to failure in producing fractalkine and neurotrophic factors such as ciliary neurotrophic factor, highlighting how this neuronal “off signal” governs the transition of microglia from homeostatic to pathogenic states (Lee et al., 2010).

These molecular switches operate at the interface between neurons and microglia, continuously sensing neuronal health and microenvironmental cues. TREM2 primarily functions as a sensor of damage-associated signals and myelin debris, directing microglial responses to tissue injury; CX3CR1 transduces neuronal-derived fractalkine signals that restrain microglial activation. Together, they form an integrated checkpoint network governing transition between homeostatic, pro-inflammatory, and anti-inflammatory states. Dysregulation of any of these switches can tip the balance toward pathogenic microglial activation, contributing to neuroinflammation and tissue damage in MS and NMOSD.

**Figure 2:**
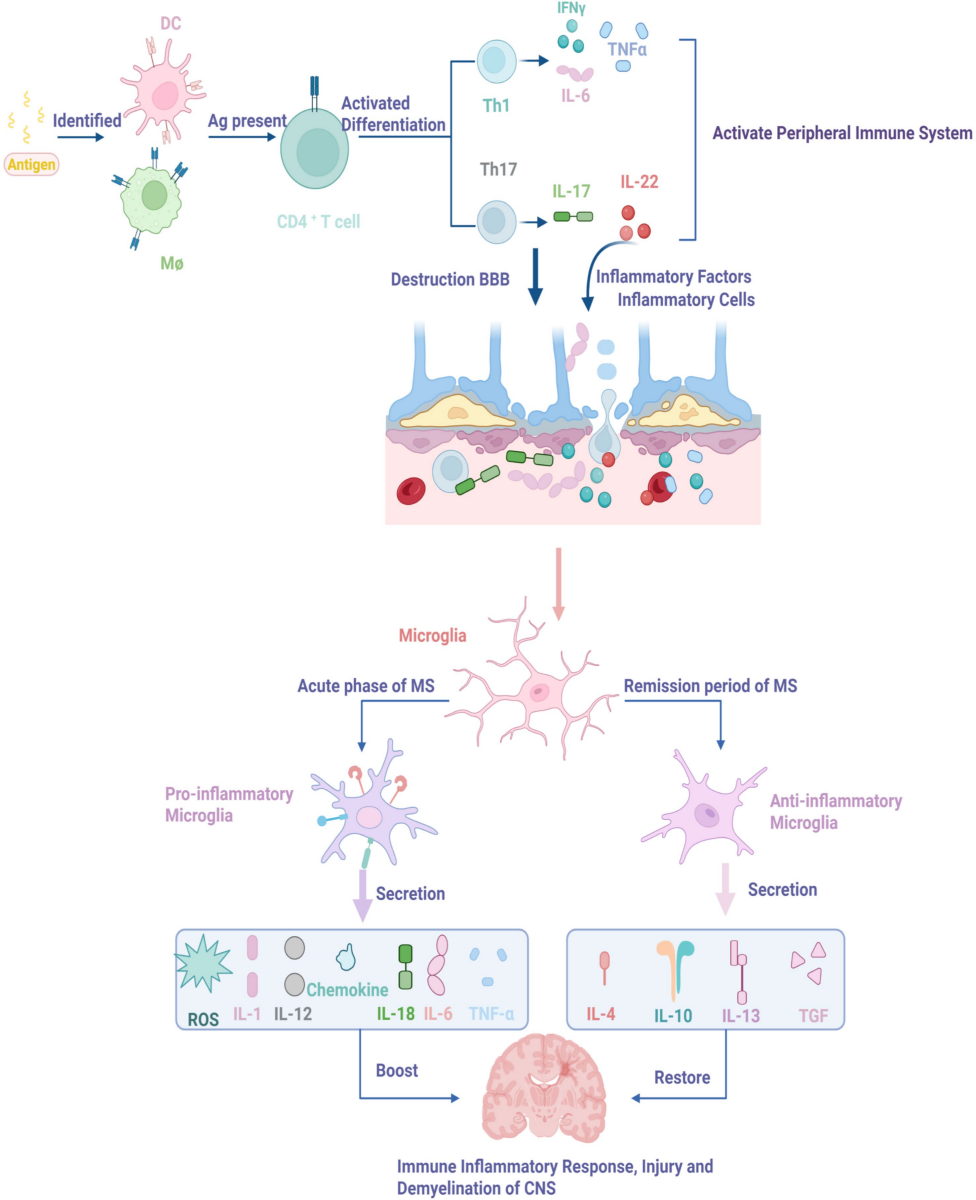
Mechanisms of peripheral immune system activation and CNS injury in the pathogenesis of MS. 1. Antigen recognition and T-cell activation: Antigen presentation activates CD4+ T cells, driving their differentiation into Th1 and Th17 effector subsets. Th1 cells secrete pro-inflammatory cytokines such as IFN-γ, TNF-α, and IL-6, which contribute to disruption of the BBB. Th17 cells release inflammatory mediators, including IL-22, amplifying the immune response. 2. Microglial polarization: During the acute phase of MS, pro-inflammatory microglia dominate and release multiple inflammatory cytokines. In the remission phase, anti-inflammatory microglia become activated and secrete immunosuppressive factors such as IL-4 and IL-10.

During remission, lesion areas still harbor abundant microglia, but these now adopt an anti-inflammatory phenotype. They secrete anti-inflammatory cytokines (e.g., IL-4, IL-10, IL-13) and growth factors (e.g., IGF-1), inhibit Th1-driven inflammation, and promote Th2 and regulatory T cell (Treg) differentiation (Zia et al., 2020; Vasileiou and Fitzgerald, 2023), thereby supporting neuronal repair, remyelination, and immunoregulation, collectively attenuating MS progression (Cao and He, 2013; Bogie et al., 2014). Furthermore, anti-inflammatory microglia phagocytose inhibitory myelin debris, secrete factors that promote remyelination, and facilitate recruitment and differentiation of oligodendrocyte precursor cells into myelinating oligodendrocytes, as depicted in Figure 2 (Vasileiou and Fitzgerald, 2023). Studies in EAE models confirm that intrathecal delivery of IL-4 induces anti-inflammatory microglia polarization, alleviating inflammation, reducing disease severity, and shortening duration (Butovsky et al., 2006). Another study showed that enhancing myelin debris clearance using neutrophil-derived nanovesicles improves microglial phagocytic function, attenuates CNS inflammation, and improves disease outcomes in EAE mice (Shen et al., 2022).

In summary, MS is a chronic inflammatory demyelinating disease involving both peripheral and central immune responses. Microglia, as CNS sentinels, play a dual role: their pro-inflammatory phenotype drives neural damage during acute phases, while their anti-inflammatory phenotype promotes repair during remission. This dynamic balance is tightly regulated by a multilayered network encompassing transcription factors (NF-κB/STAT1 vs. STAT6/PPAR-*γ*), metabolic reprogramming (glycolysis vs. oxidative phosphorylation, orchestrated by mTOR), and epigenetic modifications (histone acetylation by HDACs, and non-coding RNAs such as miR-155 and miR-124). Superimposed on these intracellular networks are critical membrane-bound molecular switches—TREM2, CX3CR1—that continuously sense neuronal health and microenvironmental cues, governing microglial transitions between functional states. The PD-1/PD-L1 pathway acts as a critical molecular switch that integrates with these regulatory networks to modulate microglial polarization and function. By regulating both T cell responses and direct microglial polarization through STAT1/NF-κB and Akt/mTOR pathways (Zhang Q. et al., 2025), PD-1/PD-L1 signaling plays a key role in limiting excessive inflammation and promoting tissue recovery, making it a promising therapeutic target. Future studies integrating single-cell multi-omics approaches will be essential to decipher the intricate crosstalk between these regulatory layers and their implications for MS pathogenesis and therapy.

### 2.4 Regulatory effects of PD-1/PD-L1 expressed by other immune cells on microglia in MS

Multiple immune cell lineages contribute to the inflammatory cascade in MS, including astrocytes, neutrophils, and B lymphocytes, each with its own specificity. Under inflammatory stimulation, PD-L1 expression increases on these cells, but they play distinct roles in modulating the response.

Immunoregulatory role of astrocytes. In MS, PD-L1 is also expressed on the surface of astrocytes in response to inflammatory factors. Engagement of PD-1 on CD8 + T cells by astrocytic PD-L1 inhibits IFN-*γ* and IL-2 production, reduces granzyme B release, and suppresses CNS inflammation. Relative to microglia, however, astrocytic PD-L1 upregulation is delayed and quantitatively weaker (Linnerbauer et al., 2023; Smith et al., 2023).

Immunomodulatory effects of neutrophils. In early MS, neutrophils exhibit an anti-inflammatory phenotype with high PD-L1 expression. Interaction with PD-1 on lymphocytes curbs Th1 cell proliferation. Neutrophils also secrete IL-10, supporting inflammation resolution. During acute phases, however, neutrophils shift to a pro-inflammatory state with reduced PD-L1, releasing ROS and MMP-9 that exacerbate tissue injury (Hu et al., 2025).

Immunomodulation of B lymphocytes. In early MS, B lymphocytes migrate from the periphery across the BBB into the CNS, guided by the CXCL13/CXCR5 axis. By expressing PD-L1, they engage PD-1 on T cells, inhibiting T-cell proliferation and reducing infiltration of peripheral inflammatory cells. In addition, B lymphocytes secrete the anti-inflammatory cytokine IL-10, which inhibits pro-inflammatory microglia polarization and further limits CNS damage (Yadav et al., 2015; Zhang et al., 2015).

Notably, an autoregulatory loop fine-tunes PD-L1 expression: excessive PD-L1 on microglia upregulates miR-146a, which in turn suppresses PD-L1 translation, maintaining physiological levels of this checkpoint molecule (Chauhan and Lokensgard, 2019; Mi et al., 2021; Bhaumik et al., 2009). In addition, emerging evidence suggests that PD-L1 may also exert transcellular regulatory effects. It has been proposed that when microglial PD-L1 expression becomes excessive, it could potentially down-regulate PD-L1 expression in astrocytes, B lymphocytes, and other cells, contributing to the stability of the anti-inflammatory environment (Chauhan and Lokensgard, 2019). However, this transcellular regulation remains incompletely understood and warrants further investigation in relevant *in vivo* models.

Exploiting the polarization plasticity of microglia, modulating the PD-1/PD-L1 pathway, offers a theoretical basis for developing therapeutic strategies to delay MS progression. However, challenges remain, including unclear downstream mechanisms and insufficient evidence for specific regulatory nodes, which urgently need to be addressed through single-cell sequencing, in vivo imaging, and other advanced technologies.

## 3 Immunopathogenesis and immunomodulation in NMOSD: a microglial perspective

### 3.1 Core pathological mechanisms of NMOSD

NMOSD is an autoimmune CNS disorder clinically characterized by optic neuritis and myelitis. Its pathological hallmarks include: (1) astrocytic injury with marked downregulation of aquaporin-4 (AQP4) and disruption of the glial fibrillary acidic protein network; (2) complement activation leading to widespread deposition of membrane attack complexes; (3) extensive demyelination; and (4) compromise of BBB integrity (Li et al., 2021).

The central pathogenic event is the production of autoantibodies against AQP4 (AQP4-IgG) (Yadav et al., 2015). AQP4 is a water-selective channel protein expressed on astrocyte end-feet, predominantly in meninges, spinal cord, and optic nerves (Howe et al., 2014; Guo et al., 2017; Saadoun et al., 2010). It plays crucial roles in maintaining water/electrolyte balance, facilitating astrocytic transport, modulating neuronal excitability, and supporting BBB metabolic functions (Cruz et al., 2021; Coutinho Costa et al., 2023). The M23 isoform of AQP4 forms orthogonal arrays on the cell membrane, serving as binding sites for AQP4-IgG (Rossi et al., 2012; Levin et al., 2013). AQP4-IgG binds specifically to AQP4 on astrocytic end-feet, either via transcytosis across endothelial cells or through a compromised BBB, forming immune complexes, downregulating AQP4 expression, and inducing astrocytes to produce and release IL-6. IL-6 further disrupts the BBB, promotes differentiation of naive T cells into Th17 cells, drives B cell differentiation into plasma cells, and stimulates further AQP4-IgG production, amplifying the inflammatory cascade (Huang et al., 2022; Fujihara et al., 2020). Of direct relevance to the PD-1/PD-L1 axis, IL-6 has been shown to upregulate PD-L1 expression on antigen-presenting cells, including microglia, through STAT3 signaling, suggesting that the initial inflammatory milieu in NMOSD may simultaneously prime immune checkpoint machinery to counterbalance excessive neuroinflammation (Zhang Q. et al., 2025; Manenti et al., 2022).

### 3.2 Dynamic polarization of microglia mediates immunomodulation in NMOSD

Microglia play a dual role in NMOSD immune regulation, polarizing toward either pro-inflammatory or anti-inflammatory phenotypes over the disease course (You et al., 2023).

In the acute phase, peripherally derived AQP4-IgG crosses the compromised BBB and binds specifically to AQP4 on astrocytes (Howe et al., 2014). This triggers complement system activation via the classical pathway, generating multiple complement components and inflammatory mediators (Bogie et al., 2014; Butovsky et al., 2006). Critically, complement activation products, particularly the anaphylatoxins C3a and C5a, serve as key signals driving microglial phenotypic polarization (Song et al., 2017; Möller et al., 1997). C3a and C5a bind to their respective receptors, C3aR and C5aR, which are constitutively expressed on microglia (Li et al., 2021; Rossi et al., 2012; Liddelow et al., 2017). Receptor engagement activates intracellular signaling cascades, including mitogen-activated protein kinase (MAPK) and NF-κB pathways, promoting polarization toward a pro-inflammatory phenotype (Li et al., 2021; Nytrova et al., 2014; Davoust et al., 1999). This shift is characterized by secretion of pro-inflammatory cytokines (e.g., IL-1β, IL-6, TNF-α) and chemokines, which recruit peripheral immune cells and amplify neuroinflammation. However, microglial responses are not uniform across the lesion; spatial transcriptomic analyses suggest that microglia at the lesion edge may exhibit a mixed phenotype, with concurrent expression of inflammatory and tissue-repair genes (Chen et al., 2021; Zrzavy et al., 2017). Moreover, activated microglia themselves produce additional complement components, including C1q and C3, establishing a positive feedback loop that sustains and amplifies the inflammatory response (Asavapanumas et al., 2021; Xu L. et al., 2023; Yick et al., 2018).

In parallel, complement-opsonized debris and immune complexes engage Fcγ receptors and complement receptors (C3R, C4R) on microglia, enhancing their phagocytic activity and further contributing to tissue injury through release of reactive oxygen species and proteolytic enzymes (Xu L. et al., 2023; Fonseca et al., 2017; Felix et al., 2016). The terminal phase of complement activation leads to assembly of the membrane attack complex (MAC/C5b-9), which forms transmembrane pores in astrocytes and other glial cells, directly causing cell lysis and tissue damage (Cardona et al., 2018; Zia et al., 2020; Smith et al., 2023). This process, together with the ongoing inflammatory cascade, establishes a self-amplifying cycle of injury.

**Figure 3:**
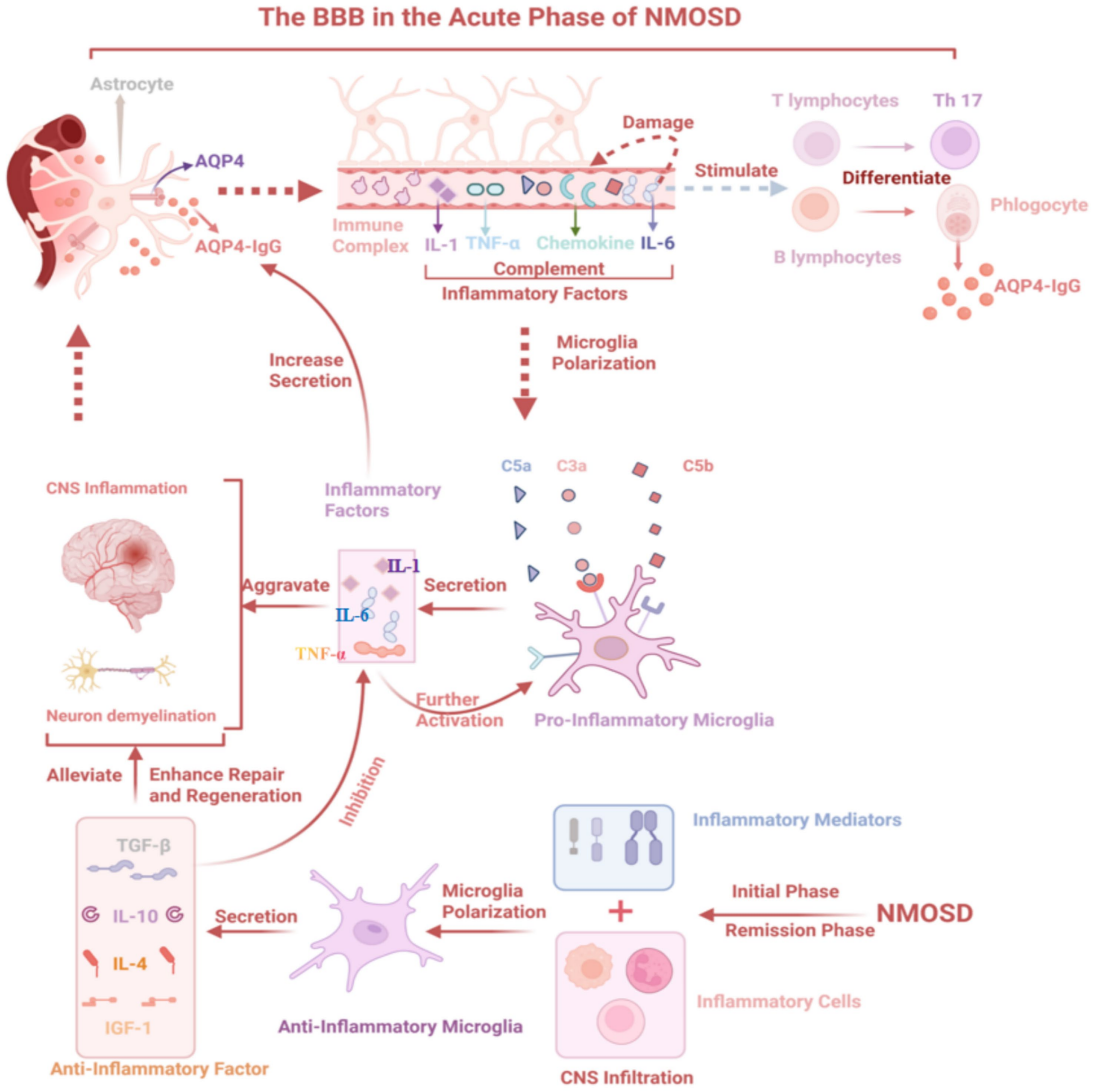
Dynamic evolution of pathological mechanisms in the acute and remission/early phases of NMOSD. Acute phase of NMOSD: peripherally activated AQP4-IgG specifically binds to AQP4 on astrocyte end-feet at the BBB, leading to BBB disruption and widespread deposition of AQP4-IgG within the CNS. The formation of immune complexes between AQP4-IgG and AQP4 channels on astrocytes activates the complement system and recruits additional inflammatory cells. Pro-inflammatory factors (e.g., IL-1, TNF-α, IL-6, complement components) and damage-associated signals drive microglial polarization toward a pro-inflammatory phenotype. These activated microglia further secrete large quantities of inflammatory mediators, establishing a positive feedback loop that exacerbates BBB disruption, oligodendrocyte injury, and demyelination. Remission/early phase of NMOSD: as the inflammatory environment subsides, anti-inflammatory signals (such as TGF-β, IL-10, and IL-4) promote the polarization of microglia toward an anti-inflammatory phenotype. These anti-inflammatory microglia secrete neurotrophic factors, including IGF-1, which contribute to suppression of inflammation and facilitate tissue repair and remyelination.

In early or remission stages of NMOSD, the microglial population shifts toward a greater prevalence of anti-inflammatory phenotypes. These cells produce cytokines, such as IL-4, IL-10, IL-13, and TGF-β, which suppress pro-inflammatory cytokine production and promote tissue repair (Liu et al., 2018; Li et al., 2021). Yet, even during remission, some pro-inflammatory microglia may persist in perilesional areas, contributing to the risk of relapse or ongoing subtle injury (You et al., 2023). Through IGF-1 secretion, microglia also promote the differentiation of oligodendrocyte precursor cells, supporting remyelination and axonal repair, as shown in Figure 3 (Xu T. et al., 2023).

The balance between pro-inflammatory and anti-inflammatory microglia phenotypes is thus not a binary switch but a dynamic equilibrium influenced by complement-derived signals, spatial location, and disease stage. While C3a and C5a drive pro-inflammatory polarization through C3aR and C5aR engagement, regulatory cytokines and anti-inflammatory mediators promote the alternative phenotype. This dynamic equilibrium determines the trajectory of neuroinflammation and tissue damage in NMOSD. Critically, the PD-1/PD-L1 axis intersects with this balance at multiple levels: PD-L1 engagement on microglia has been shown to suppress NF-κB activation, a key downstream effector of C5aR signaling, potentially raising the threshold for complement-driven pro-inflammatory polarization (Zhang Q. et al., 2025; Manenti et al., 2022). Conversely, pro-inflammatory microglia upregulate PD-L1 as a negative feedback mechanism, suggesting that the extent of complement activation may directly influence the capacity of microglia to restrain T cell responses via PD-1/PD-L1 interactions (Chauhan and Lokensgard, 2019; Liu et al., 2018).

A comprehensive summary of the roles of individual complement components in microglial activation and CNS injury is provided in Table 1, highlighting the central position of microglia as both sensors and effectors of complement-mediated pathology.

**Table 1:** Summary of mechanisms by which complement system components regulate microglial functions and mediate central nervous system injury.

Complement components	Source/activation pathway	Receptors/binding target	Cellular effects	Downstream pathological consequences	References
C1q	Classical pathway (AQP4-IgG-Fc activated)	AQP4 + IgG Fc segment of astrocytes	❖Initiating the classical complement cascade (C4 → C2 → C3 convertase) ❖Induces microglial secretion of C1q and IL-6	✤Positive feedback activation of complement cascade ✤Pro-inflammatory microglial polarization	Li et al. (2021), Rossi et al. (2012); Levin et al. (2013), and Liddelow et al. (2017)
C3a	Generated by C3 convertase cleavage of C3	Microglial C3aR	❖Recruits inflammatory cells into CNS ❖Promotes pro-inflammatory microglia polarization and secretion of IL-6 and other factors	✤Worsening of CNS inflammatory microenvironment ✤Oligodendrocyte apoptosis ✤Impaired myelination	Li et al. (2021), Nytrova et al. (2014), and Davoust et al. (1999)
C3b	Generated by C3 convertase cleavage of C3	Astrocyte surface + microglia CR1 (CD35)	❖Enhances phagocytosis via opsonization ❖Forms C5 convertase	✤Astrocyte injury ✤BBB disruption ✤Amplification of inflammatory response	Asavapanumas et al. (2021) and Xu L. et al. (2023)
C4	Generated by C1 complex cleavage of C4	C4b binds to cell surface	❖C4b and C2a form C3 convertase ❖C4a (weak anaphylatoxin) increases vascular permeability	✤Promotes C3 cleavage → amplifies complement activation ✤BBB disruption	Pache et al. (2021)
C5a	Generated by C5 convertase cleavage of C5	Microglia C5aR(CD88)	❖Disrupts BBB ❖Amplifies CNS inflammatory response ❖Glutamate transport dysfunction → neurotoxicity to neurons/oligodendrocytes ❖Interference with myelination	✤Excitotoxic neuronal death ✤Oligodendrocyte injury and demyelination ✤Demyelination	Fonseca et al. (2017) and Araki (2019)
C5b	Generated by C5 convertase cleavage of C5	Forms the MAC with C6-C9, inserting into the cell membrane	❖Induces cell lysis and secondary inflammation	✤Astrocyte damage ✤BBB disruption ✤Secondary neuronal damage	Fonseca et al. (2017) and Araki (2019)

This table systematically summarizes the sources, interactions, and core pathological functions of key complement components in NMOSD. The binding of AQP4-IgG to AQP4 on astrocytic surfaces activates the classical complement pathway, triggering a cascade that leads to core pathological processes including astrocyte injury, BBB disruption, amplification of inflammation, and demyelination. As pivotal immune effector cells within the CNS, microglia express various complement receptors such as C3aR, C5aR, and C3R/C4R—through which they are activated by complement components and polarized toward a pro-inflammatory phenotype. This activation promotes the secretion of inflammatory factors, including IL-6, significantly exacerbating neuroinflammation and tissue damage. The interaction between the complement system and microglia constitutes a critical positive feedback loop in the progression of NMOSD.

### 3.3 Microglial involvement in NMOSD immunoregulation via the PD-1/PD-L1 axis

The PD-1/PD-L1 immune checkpoint plays a key immunomodulatory role in NMOSD pathogenesis by negatively regulating immune responses. Direct evidence from patient-based studies supports the involvement of this pathway in NMOSD (Guo et al., 2022). A study of 30 NMOSD patients demonstrated that expression levels of membrane-bound PD-1 on peripheral CD4^+^ T cells, as well as PD-L1 on CD14^+^ monocytes and CD19^+^ B cells, were significantly elevated compared to healthy controls and disease control groups [longitudinally extensive transverse myelitis (LETM), optic neuritis (ON), and other CNS diseases] (Zhang et al., 2022; Chen et al., 2012; Xue et al., 2019) Furthermore, serum levels of soluble PD-1 (sPD-1) and soluble PD-L1 (sPD-L1) were also significantly increased in NMOSD patients, suggesting systemic upregulation of this inhibitory pathway in response to active neuroinflammation (Zhang et al., 2022; Chen et al., 2012).

Regarding cellular localization relevant to the CNS, microglia express PD-L1 upon activation, positioning them to interact with PD-1-expressing T cells that infiltrate the CNS during NMOSD exacerbations. While direct immunohistochemical evidence of microglial PD-L1 expression in NMOSD lesions remains limited, the observed upregulation of PD-L1 on peripheral monocytes, which share a common myeloid lineage with microglia, supports the concept that similar regulatory mechanisms operate within the CNS compartment (Zhang et al., 2022; Xue et al., 2019).

The correlation between PD-1/PD-L1 expression and disease activity provides further insight into their functional relevance. One study found that unbalanced expression of ICOS (a positive costimulatory molecule) and PD-1 (a negative costimulatory molecule) in NMOSD patients may contribute to immune dysregulation, with the ICOS/PD-1 ratio potentially serving as a biomarker for early-stage NMOSD diagnosis (Zhang et al., 2022; Xue et al., 2019). Additionally, a recent study examining coinhibitory receptors in NMOSD observed alterations in multiple inhibitory pathways, suggesting a complex regulatory network in which PD-1/PD-L1 signaling participates alongside other checkpoint molecules (Haham et al., 2024).

Perhaps the most compelling evidence for the functional significance of PD-1/PD-L1 signaling in NMOSD comes from clinical observations of immune checkpoint inhibitor (ICI) therapy. Multiple case reports have documented the development of *de novo* AQP4-IgG-positive NMOSD following treatment with PD-1/PD-L1 inhibitors. In one reported case, a patient with lung adenocarcinoma developed anti-AQP4 antibody-positive myelitis after a single cycle of pembrolizumab (a PD-1 monoclonal antibody) (Shimada et al., 2020). Analysis of cerebrospinal fluid revealed a transient increase in plasmablasts, supporting the hypothesis that PD-1 blockade may unleash not only T-cell responses but also B-cell-mediated autoimmunity, leading to AQP4 antibody production and CNS demyelination (Shimada et al., 2020). This clinical observation provides reverse translational evidence that PD-1/PD-L1 signaling normally functions to restrain pathogenic autoimmune responses targeting AQP4. Further supporting this concept, a paraneoplastic NMOSD case involving esophagogastric junction adenocarcinoma revealed an intriguing relationship between tumoral PD-L1 expression and AQP4-IgG production. In this case, tumor cells expressing AQP4 were negative for PD-L1 staining, suggesting that the absence of PD-L1-mediated immune evasion allowed immune surveillance to recognize “unprotected” AQP4 as a neoantigen, triggering cross-reactive AQP4-IgG production and NMOSD (Sudo et al., 2018). This pathological observation underscores the critical role of PD-L1 in maintaining immune tolerance to AQP4 and preventing autoimmune targeting of the CNS.

Collectively, these findings suggest that the PD-1/PD-L1 pathway plays a critical immunoregulatory role in NMOSD. During antigen presentation, PD-1/PD-L1 engagement suppresses T cell effector functions, including production of pro-inflammatory cytokines, such as IFN-*γ*, while promoting secretion of immunosuppressive cytokines such as IL-10. This interaction inhibits excessive immune activation through: (1) suppression of T cell activation, clonal expansion, and effector differentiation; and (2) inhibition of B cell activation, proliferation, and antibody secretion (Guo et al., 2022; Zamani et al., 2016; Zacca et al., 2018). This feedback loop helps limit excessive neuroinflammation and protects CNS tissues from immune-mediated damage (Xue et al., 2019; Liu et al., 2021). For microglia, PD-1/PD-L1 engagement has direct functional consequences: PD-L1 signaling reduces their phagocytic activity and cytokine production, potentially limiting clearance of complement-opsonized debris and thereby modulating the inflammatory milieu (Liu et al., 2018; Li et al., 2021). PD-1-expressing microglia exposed to PD-L1^+^ astrocytes or neurons may be rendered less responsive to activating stimuli, raising the threshold for pro-inflammatory polarization and contributing to resolution of neuroinflammation (Linnerbauer et al., 2023).

The observation that PD-1/PD-L1 pathway blockade can trigger *de novo* NMOSD provides compelling evidence that this checkpoint is essential for maintaining immune tolerance to CNS autoantigens, including AQP4. Future studies should focus on characterizing PD-L1 expression on microglia within NMOSD lesions and exploring whether enhancing this pathway could represent a therapeutic strategy to restrain neuroinflammation.

### 3.4 Modulation of microglia via PD-1/PD-L1 axis on other immune cells in NMOSD

Beyond microglia, B cells and T cells also contribute significantly to NMOSD pathology. B cells participate through three major subsets: memory B cells, plasma cells, and regulatory B cells (Bregs). B cells differentiate into memory B cells and plasma cells. Memory B cells secrete IL-6, which sustains plasma cell activity and AQP4-IgG production, damages the BBB, and promotes Th17 cell differentiation, thereby exacerbating CNS injury. Plasma cells are the primary producers of AQP4-IgG; binding of AQP4-IgG to AQP4 on astrocytes is the crucial initiating event in NMOSD autoimmune injury (Häusser-Kinzel and Weber, 2019). Bregs, derived from naive, immature, or plasma B cells, secrete IL-10, IL-35, and TGF-β1, which inhibit Th1 and Th17 differentiation, reduce antigen presentation by dendritic cells and macrophages, and attenuate inflammatory cytokine secretion, thereby alleviating CNS inflammation (Yadav et al., 2015).

In the peripheral autoimmune response, B cells, functioning as antigen-presenting cells, process and present autoantigens via MHC-II and costimulatory molecules to T cells, activating antigen-specific T cell responses and influencing disease progression (Xue et al., 2019). In acute NMOSD, pro-inflammatory cytokines such as IL-6, IL-21, and TGF-β promote differentiation of naive CD4^+^ T cells into Th17 cells. Th17 cells secrete IL-17, IL-6, granulocyte macrophage colony-stimulating factor (GM-CSF), CXCL8, and other chemokines, recruiting neutrophils, macrophages, and eosinophils into CNS lesions, where they release enzymes and neurotoxins causing severe inflammation and tissue damage (Araki, 2019; Yick et al., 2023; Lin et al., 2016). Under the sole influence of TGF-β, naive CD4^+^ T cells differentiate into Tregs. Tregs secrete immunosuppressive factors, including IL-10, IL-35, perforin, granzymes, and TGF-β, promoting microglial polarization toward an anti-inflammatory phenotype. This reduces levels of chemokines and pro-inflammatory cytokines, alleviating CNS tissue damage and suppressing neuroinflammatory responses (Yang X. et al., 2021; Ma et al., 2021). Another T cell subset, T follicular helper (Tfh) cells, are stimulated by IL-12, IL-23, TGF-β, etc. During inflammation, promoting B cell activation and AQP4 antibody production, worsening NMOSD immunopathology (Yick et al., 2023).

B and T cells interact with microglia via the PD-1/PD-L1 axis, modulating NMOSD progression through several mechanisms: (1) inhibiting excessive microglial activation; (2) regulating microglial polarization toward pro−/anti-inflammatory phenotypes; and (3) balancing neuroinflammation and repair. Specifically, PD-L1 expression on B cells and Tregs can engage PD-1 on microglia, directly suppressing microglial pro-inflammatory responses and promoting a more reparative phenotype (Yadav et al., 2015; Guo et al., 2022). Conversely, PD-1-expressing T cells engaged by microglial PD-L1 are rendered less capable of providing activating signals to microglia, creating a bidirectional regulatory loop that tunes the inflammatory set point within the CNS (Chauhan and Lokensgard, 2019; Manenti et al., 2022). This cross-talk may be particularly important in NMOSD, where B cell-derived IL-6 and T cell-derived cytokines converge to shape the local microenvironment; PD-1/PD-L1 interactions thus serve as a critical rheostat integrating signals from multiple immune cell types to determine the ultimate microglial response.

Targeting key microglia-mediated mechanisms in NMOSD, such as reducing microglial activation or enhancing their phagocytic function during remission via IL-6 signaling blockade (Araki, 2019; Lotan et al., 2021), TNF-α antagonism, complement inhibition, or promoting anti-inflammatory polarization, has shown promising clinical outcomes (Chen et al., 2021; Li et al., 2021; Xu T. et al., 2023). Further investigation into microglial PD-1/PD-L interactions may provide novel insights into NMOSD immunology and inform immune checkpoint-targeted therapies.

Currently, research on the regulation of microglial polarization remains limited, particularly regarding the role of the PD-1/PD-L1 pathway. This knowledge gap is especially pertinent in NMOSD, where the complement-driven inflammatory milieu and the subsequent microglial response create a unique context for PD-1/PD-L1 regulation. Understanding how complement activation, IL-6 signaling, and B/T cell interactions converge to modulate microglial PD-L1 expression and PD-1 sensitivity will be essential for developing targeted immunotherapies that harness the full potential of this checkpoint axis. Future studies elucidating the molecular mechanisms of microglial polarization and PD-1/PD-L1-mediated immunoregulation will provide a theoretical foundation and novel therapeutic targets for diagnosis and treatment of NMOSD. The next section extends this discussion by examining how microglia interact with other immune cells through the PD-1/PD-L1 axis, comparing these interactions between MS and NMOSD.

## 4 Cross-talk between microglia and other immune cells via the PD-1/PD-L1 axis: a comparative perspective in MS and NMOSD

Beyond the direct immunomodulatory effects of the PD-1/PD-L1 axis on microglia themselves, a complex multi-cellular regulatory network exists in which various immune and glial cells interact with microglia through PD-1/PD-L1 signaling and other paracrine pathways. These interactions exhibit both commonalities and disease-specific features when comparing MS and NMOSD, reflecting the distinct immunopathological landscapes of these two CNS demyelinating disorders.

### 4.1 Astrocyte-microglia crosstalk

Astrocytes represent the most abundant glial cell population in the CNS and serve as critical regulators of neuroinflammation. In both MS and NMOSD, reactive astrocytes upregulate PD-L1 expression in response to inflammatory stimuli, positioning them to interact with PD-1-expressing immune cells, including microglia (Chauhan and Lokensgard, 2019; Smith et al., 2023).

In MS, astrocytic PD-L1 expression is induced during active neuroinflammation, particularly in perivascular regions and active demyelinating plaques. PD-1-expressing microglia have been shown to form inhibitory synapses with PD-L1^+^ astrocytes, suppressing inflammatory T cell responses and reducing infiltration of peripheral immune cells (Linnerbauer et al., 2023). This astrocyte-microglia interaction via the PD-1/PD-L1 axis represents a key endogenous mechanism for limiting CNS inflammation and promoting recovery. Relative to microglia, however, astrocytic PD-L1 upregulation is relatively delayed and weaker in magnitude, suggesting that microglia serve as the first line of immune checkpoint-mediated regulation, with astrocytes providing secondary, sustaining inhibitory signals (Chauhan and Lokensgard, 2019).

In NMOSD, the astrocyte-microglia axis assumes particular pathological significance due to the central role of AQP4-targeted autoimmunity. Astrocytes are the primary targets of AQP4-IgG-mediated injury, and their damage triggers robust complement activation and inflammatory cascades (Li et al., 2021). Reactive astrocytes in NMOSD lesions produce large quantities of IL-6, which not only disrupts the BBB but also promotes Th17 differentiation and sustains AQP4-IgG production (Huang et al., 2022; Fujihara et al., 2020). Astrocytes in NMOSD lesions also upregulate PD-L1, potentially as a compensatory anti-inflammatory response to limit ongoing tissue damage (Chauhan and Lokensgard, 2019). However, whether this astrocytic PD-L1 directly engages PD-1 on microglia in NMOSD, similar to the inhibitory synapses observed in MS, remains to be definitively established. Given that microglia in NMOSD lesions express high levels of PD-1, it is plausible that similar astrocyte-microglia inhibitory interactions occur, representing a shared immunoregulatory mechanism across both diseases.

### 4.2 B cell-microglia interactions

B cells contribute to the pathogenesis of both MS and NMOSD through distinct mechanisms, and their interactions with microglia are modulated by the PD-1/PD-L1 axis.

In MS, B lymphocytes migrate from the peripheral circulation across the BBB into the CNS, mediated by the CXCL13/CXCR5 axis. Within the CNS compartment, B cells express PD-L1 and engage PD-1 on T cells, inhibiting T cell proliferation and reducing inflammatory cell infiltration (Yadav et al., 2015; Zhang et al., 2015). Additionally, a subset of Bregs secretes IL-10, which directly inhibits pro-inflammatory microglia polarization and attenuates CNS tissue damage (Yadav et al., 2015). This B cell-mediated immunomodulation represents an important feedback loop restraining excessive neuroinflammation.

In NMOSD, B cells play a more direct pathogenic role through production of AQP4-IgG by plasma cells. Memory B cells secrete IL-6, which sustains plasma cell activity and promotes Th17 differentiation, thereby amplifying CNS injury (Liu et al., 2021). Concurrently, Bregs exert protective effects through IL-10, IL-35, and TGF-β1 secretion, inhibiting Th1/Th17 responses and promoting anti-inflammatory microglia polarization (Yadav et al., 2015). The PD-1/PD-L1 axis modulates this balance: PD-L1 expression on B cells engages PD-1 on T cells, suppressing T cell activation and indirectly influencing microglial function (Guo et al., 2022; Zacca et al., 2018). PD-1/PD-L1 signaling also directly inhibits B cell activation, proliferation, and antibody secretion, providing a checkpoint mechanism that limits AQP4-IgG production (Guo et al., 2022; Zamani et al., 2016). The observation that PD-1 blockade can trigger NMOSD underscores the critical importance of this pathway in restraining B cell-mediated autoimmunity (Shimada et al., 2020).

### 4.3 Neutrophil-microglia interactions

Neutrophils exhibit disease-specific roles in MS and NMOSD, with corresponding differences in their PD-1/PD-L1-mediated interactions with microglia.

In MS, neutrophils display phenotypic plasticity during disease progression. In early stages, they adopt an anti-inflammatory phenotype characterized by high PD-L1 expression and IL-10 secretion, which curb Th1 cell proliferation and support inflammation resolution (Manenti et al., 2022). During acute exacerbations, however, neutrophils shift to a pro-inflammatory state with reduced PD-L1, releasing ROS and MMP-9 that exacerbate tissue injury (Manenti et al., 2022). This dynamic regulation suggests that neutrophil PD-L1 expression may serve as a rheostat controlling the balance between pro- and anti-inflammatory influences on the CNS microenvironmental, indirectly modulating microglial activation states.

In NMOSD, neutrophils play a more prominent pathogenic role. Th17 cells recruit neutrophils into CNS lesions through IL-17 and GM-CSF secretion, where they release proteolytic enzymes and neurotoxins that cause severe tissue damage (Araki, 2019; Yick et al., 2023). The PD-1/PD-L1 axis modulates this process by suppressing Th17 differentiation and function. While direct evidence for neutrophil-microglia crosstalk via PD-1/PD-L1 in NMOSD remains limited, it is plausible that PD-L1-expressing neutrophils could engage PD-1 on microglia, influencing their polarization state. Conversely, neutrophil-derived inflammatory mediators likely promote pro-inflammatory microglia polarization, establishing a positive feedback loop that amplifies neuroinflammation.

### 4.4 T cell-microglia interactions and the regulatory balance

T cells represent the primary targets of PD-1/PD-L1-mediated immunoregulation, and their interactions with microglia are central to disease pathogenesis in both MS and NMOSD.

In MS, autoreactive T cells differentiate into Th1 and Th17 subsets, releasing IFN-γ, TNF-α, and IL-6 that activate microglia and recruit peripheral immune cells (Manel et al., 2008; Raphael et al., 2015; Shi et al., 2026). Microglial PD-L1 engages PD-1 on these T cells, transmitting inhibitory signals that suppress proliferation, cytokine production, and cytotoxic effector functions (Chauhan and Lokensgard, 2019; Zhao et al., 2014). This interaction is critical for limiting T cell-mediated neuroinflammation and promoting recovery. Tregs, which express high levels of PD-1, secrete IL-10 and TGF-β that promote anti-inflammatory microglia polarization (Zia et al., 2020; Vasileiou and Fitzgerald, 2023).

In NMOSD, Th17 cells play a particularly prominent role, secreting IL-17, IL-22, and GM-CSF that recruit neutrophils and activate microglia (Araki, 2019; Yick et al., 2023). Tregs exert protective effects by suppressing Th17 responses and promoting anti-inflammatory microglial polarization through IL-10 and TGF-β secretion (Yang X. et al., 2021; Ma et al., 2021). The PD-1/PD-L1 axis modulates this balance by inhibiting Th17 differentiation and function while promoting Treg development and activity (Zhao et al., 2014; Zamani et al., 2016). Tfh cells, which promote B cell activation and AQP4 antibody production, are also regulated by PD-1 signaling, with PD-1 deficiency leading to enhanced Tfh responses and exacerbated autoimmunity (Yick et al., 2023).

### 4.5 Transcellular regulation and feedback loops

An additional layer of complexity involves transcellular regulation of PD-L1 expression across different cell types. When microglial PD-L1 expression becomes excessive, it upregulates miR-146a (Yang et al., 2012), which not only suppresses PD-L1 translation in microglia through negative feedback but may also downregulate PD-L1 expression in astrocytes and B lymphocytes (Chauhan and Lokensgard, 2019; Manel et al., 2008; Yang X. et al., 2021). This transcellular regulatory mechanism helps maintain the stability of the anti-inflammatory environment and prevents excessive immunosuppression that could impair pathogen defense.

### 4.6 Comparative summary: disease-specific features of microglial involvement in MS and NMOSD

In summary, although MS and NMOSD are both CNS demyelinating diseases, their immunopathological mechanisms are fundamentally distinct. MS is primarily T cell-mediated, with microglial polarization driven by T cell-derived cytokines; the PD-1/PD-L1 axis predominantly targets T cells, and complement involvement is limited. In contrast, NMOSD is centered on AQP4-IgG-mediated humoral immunity, with complement activation products C5a and C3a serving as key drivers of pro-inflammatory microglia polarization. The PD-1/PD-L1 axis in NMOSD not only regulates T cells but also directly inhibits B cell activation and AQP4-IgG production, positioning the complement system as a core pathogenic player. These differential insights suggest that therapeutic strategies for MS should focus on the T cell-microglia axis, whereas NMOSD treatment necessitates simultaneous targeting of the complement cascade, IL-6 signaling, and B cell-microglia crosstalk, with combination approaches offering potential synergistic benefits.

## 5 Current research limitations and future perspectives

### 5.1 Current research limitations

The dynamic changes in microglial polarization across different stages of MS and NMOSD—along with the precise molecular mechanisms governing the transition from a pro-inflammatory to an anti-inflammatory phenotype—remain poorly elucidated. Current research has primarily focused on the PD-1/PD-L1 functions of T and B cells, whereas regulation of PD-1/PD-L1 expression in microglia and its impact on neuroinflammation require further exploration. In particular, how the PD-1/PD-L1 axis precisely modulates this process demands in-depth investigation.

Most existing studies on PD-1/PD-L1 signaling in CNS demyelinating diseases rely on animal models, particularly EAE (Linker and Lee, 2009). While EAE has been instrumental in elucidating basic mechanisms of T cell-mediated autoimmunity and testing immunomodulatory therapies, it has significant limitations in recapitulating the full complexity of human MS and NMOSD. EAE is primarily a CD4^+^ T cell-driven model, whereas MS involves both CD4^+^ and CD8^+^ T cells, B cells, and innate immune contributions. More critically, EAE does not replicate the humoral and complement-mediated pathology characteristic of NMOSD, where AQP4-IgG binding to astrocytes triggers complement activation and downstream inflammatory cascades (Capper et al., 2025). Although AQP4-IgG passive transfer models have been developed to study NMOSD pathogenesis, these models often involve acute, high-dose antibody administration and may not fully capture the chronic, relapsing nature of the human disease or the complex interplay between peripheral immune tolerance breakdown and CNS-compartmentalized inflammation (Duan and Verkman, 2020). Furthermore, species-specific differences in immune cell phenotypes, cytokine responses, and PD-1/PD-L1 regulation between rodents and humans limit the direct translatability of findings from animal models to clinical applications (Carter et al., 2007).

Importantly, the clinical relevance of the PD-1/PD-L1 axis in CNS demyelinating diseases is underscored by real-world observations from oncology practice. A systematic literature review identified 23 cases of CNS demyelination associated with ICI therapy, including seven with myelitis, four with isolated optic neuritis, one with NMOSD, five with MS, and six with atypical demyelination (Oliveira et al., 2020). The median time to symptom onset was 6.5 weeks from ICI initiation, with a median of 14 days from the last ICI dose. Notably, outcomes were generally favorable after immunosuppression, with 18 patients showing improvement or full recovery (Oliveira et al., 2020). A separate study analyzing 422 consecutive patients at a tertiary neuroimmunology clinic over 5 years found that iatrogenic CNS inflammation accounted for 6.4% of new referrals, with ICIs implicated in 26% of these cases (Kelly et al., 2022). The clinical phenotypes included MS relapses (37%), autoimmune encephalitis (30%), NMOSD attacks (15%), and transverse myelitis (11%) (Kelly et al., 2022).

More specifically, multiple case reports have documented AQP4-IgG-positive NMOSD following PD-1/PD-L1 inhibitor treatment (Shimada et al., 2020; Hirano et al., 2022). In one case, a patient with lung adenocarcinoma developed anti-AQP4 antibody-positive myelitis after a single cycle of pembrolizumab, with cerebrospinal fluid analysis revealing a transient increase in plasmablasts, supporting the hypothesis that PD-1 blockade unleashes not only T-cell responses but also B-cell-mediated autoimmunity (Shimada et al., 2020). A case of NMOSD without AQP4-IgG precipitated by nivolumab suggests a possible T-cell mediated pathogenesis in seronegative patients, offering insights into why this patient subgroup may lack response to B-cell therapies in clinical trials (Nasralla and Abboud, 2020). These clinical observations provide reverse translational evidence that tonic PD-1/PD-L1 signaling is essential for maintaining immune tolerance to CNS autoantigens, and that its disruption can trigger the very diseases that are the focus of this review.

Beyond the limitations of individual models, the reductionist approach of studying isolated cell populations or pathways in controlled experimental settings may overlook the dynamic, multi-cellular interactions and spatial heterogeneity that characterize human neuroinflammatory lesions. For example, the role of microglial PD-L1 in promoting T cell exhaustion observed in chronic viral models has not been rigorously validated in human MS or NMOSD tissues (Prasad et al., 2017). Similarly, the therapeutic window for PD-1/PD-L1 modulation, balancing suppression of pathogenic autoimmunity against preservation of protective immune surveillance, cannot be accurately defined solely from animal studies.

Moreover, systemic modulation of the PD-1/PD-L1 axis may trigger immune-related adverse effects, including autoimmune activation. Therefore, achieving CNS-specific targeting remains a critical challenge for therapeutic development. These considerations underscore the urgent need for complementary approaches—including human tissue studies, induced pluripotent stem cell-derived microglial models, and careful integration of clinical cohort data with multi-omics analyses—to validate and extend findings from animal models and guide rational design of next-generation immunotherapies.

### 5.2 Future perspectives: translational opportunities and challenges

Future studies should integrate single-cell sequencing, spatial transcriptomics, and epigenomics to dissect the regulatory networks linking PD-1/PD-L1 to microglial polarization. For instance, single-cell RNA sequencing of MS lesions could deconvolute disease-stage-specific microglial subpopulations and identify those most responsive to PD-1/PD-L1 modulation (Liu et al., 2025; Chen Z. et al., 2025). Spatial transcriptomics could map PD-1^+^ T cells and PD-L1^+^ microglia within active plaques, revealing the spatial dynamics of immune checkpoint interactions. Complementary single-cell ATAC-seq would elucidate epigenetic mechanisms governing microglial polarization and PD-L1 expression, pinpointing targetable transcription factors.

Satralizumab, a humanized monoclonal antibody targeting the IL-6 receptor (IL-6R), has demonstrated robust efficacy in preventing NMOSD relapses in both pivotal clinical trials and real-world settings (Greenberg et al., 2025; Lin et al., 2025). Real-world data from the TriNetX platform showed that satralizumab was associated with significantly lower relapse risk compared to conventional immunosuppressants across all follow-up intervals up to 36 months, with numbers needed to treat consistently between 4 and 9 (Lin et al., 2025). Importantly, satralizumab treatment permits dose reduction of concomitant oral glucocorticoids and immunosuppressants, with 71.3% of patients achieving glucocorticoid dose reduction by 26 weeks in a Japanese real-world study (Fujihara et al., 2025). Safety analyses indicate that infection rates with satralizumab are consistently lower than background infection rates in NMOSD patients from real-world data, supporting its favorable risk–benefit profile (Greenberg et al., 2025).

Given that the PD-1/PD-L1 pathway intersects with both IL-6 signaling and complement activation at multiple levels, combination strategies warrant systematic investigation. Mechanistically, IL-6 has been shown to promote T cell exhaustion and upregulate PD-1 expression on CD8^+^ T cells in various disease contexts (Zhang L. et al., 2025). In non-small cell lung cancer models, combined blockade of IL-6 and PD-1 substantially enhanced the effector function of CD8^+^ T cells and inhibited tumor growth, suggesting potential synergy between these pathways (Zhang L. et al., 2025). Translating this concept to CNS demyelinating diseases, one could hypothesize that combining PD-1/PD-L1 agonists with IL-6R blockade (e.g., satralizumab) might provide complementary immunomodulatory effects: IL-6R inhibition targets the pro-inflammatory milieu that drives Th17 differentiation and AQP4-IgG production, while PD-1/PD-L1 agonism directly suppresses T cell activation and promotes regulatory T cell function. The optimal sequencing of such combination therapy—whether concurrent administration or sequential addition of PD-1/PD-L1 agonists after achieving disease control with IL-6R blockade—remains to be determined through preclinical modeling and carefully designed clinical trials.

CNS-targeted delivery (e.g., nanocarriers) may mitigate systemic immune-related adverse events, as neurological irAEs occur in 6–12% of patients on systemic checkpoint inhibitors (Manenti et al., 2022). Real-world data from eculizumab cohorts underscore that even effective therapies carry risks (e.g., 13% serious infections, 10% mortality in older disabled patients), necessitating careful risk–benefit assessment. Bridging basic insights with clinical translation will pave the way for next-generation immunotherapies in CNS demyelinating diseases (Tao et al., 2025).

## 6 Conclusion

This review systematically elaborates the immunomodulatory mechanisms underlying MS and NMOSD, two classic CNS demyelinating diseases, with a particular emphasis on the dual role of microglia throughout disease progression and their critical function in maintaining immune homeostasis and regulating neuroinflammation via the PD-1/PD-L1 axis. Elucidating the dynamic polarization of microglia across disease stages and within distinct lesion compartments is essential not only for understanding the pathogenesis of MS and NMOSD, but also for providing novel perspectives on the immunology of CNS demyelinating disorders. Recognizing that microglial phenotypes exist along a continuum and exhibit spatial heterogeneity will inform more precise therapeutic strategies aimed at modulating microglial function in a stage- and region-specific manner.

An emerging and clinically relevant dimension of this regulatory axis involves T cell exhaustion, a state of progressive dysfunction characterized by sustained inhibitory receptor expression and diminished effector function (Wherry and Kurachi, 2015). Chronic neuroinflammation in progressive MS and NMOSD creates an environment conducive to T cell exhaustion, yet direct evidence within the CNS compartment remains limited. Single-cell studies of CSF from progressive MS patients have identified clonally expanded CD8^+^ T cells with exhaustion-associated transcriptional signatures, suggesting that chronic antigen stimulation may drive T cells toward a hyporesponsive state (McLane et al., 2019; Pauken and Wherry, 2015). Microglia, through their constitutive and IFN-*γ*-inducible PD-L1 expression, are uniquely positioned to orchestrate this process. By engaging PD-1 on infiltrating T cells, microglial PD-L1 may actively promote and maintain T cell exhaustion within the CNS, limiting immunopathology while potentially compromising beneficial immune surveillance. The intersection of this exhaustion axis with other microglial regulatory pathways—including TREM2 and CX3CR1 signaling—adds further complexity and represents an important frontier for future investigation.

Based on the evidence reviewed herein, we propose the following testable hypotheses to guide future investigations: (1) In MS, enhancing PD-L1 expression on microglia during the acute phase promotes their transition from a pro-inflammatory to an anti-inflammatory phenotype, thereby attenuating neuroinflammation and improving clinical outcomes. This hypothesis can be tested using EAE models with microglia-specific PD-L1 overexpression or conditional knockout strategies. (2) In NMOSD, the PD-1/PD-L1 axis acts as a critical checkpoint that counter-regulates complement-driven microglial activation. Specifically, we hypothesize that PD-L1 engagement on microglia suppresses C5a receptor-mediated pro-inflammatory polarization, and that pharmacological activation of this pathway during remission phases may enhance microglial phagocytic clearance of myelin debris and promote remyelination. This could be validated using *in vitro* microglial cultures exposed to NMOSD patient sera or complement components, combined with PD-1/PD-L1 agonists or blocking antibodies.

Critically, the relationship between microglial PD-L1 and T cell exhaustion raises fundamental questions that warrant urgent investigation: Do exhausted T cells within MS and NMOSD lesions retain the capacity for reactivation, and if so, what triggers this? Can microglial PD-L1 expression be therapeutically modulated to rebalance the immune response—promoting exhaustion of pathogenic T cells while preserving protective immunity? How do existing disease-modifying therapies intersect with this exhaustion axis? Addressing these questions through integration of single-cell technologies, functional assays using human CNS tissue, and careful preclinical modeling will be essential for translating our understanding of the PD-1/PD-L1 axis into precisely targeted immunotherapies.

Testing these hypotheses will not only deepen our understanding of microglial biology and the PD-1/PD-L1 pathway but may also facilitate the development of more effective and precise immune checkpoint-targeted therapies for CNS demyelinating diseases.

## Glossary

AQP4: aquaporin-4
AQP4-IgG: aquaporin-4 immunoglobulin G
ATAC-seq: assay for transposase-accessible chromatin using sequencing
BBB: blood–brain barrier
Bregs: regulatory B cells
C/EBP-*α*: CCAAT/enhancer-binding protein α
C1q: complement component 1q
C3: complement component 3
C3a: complement component 3a
C3aR: C3a receptor
C3b: complement component 3b
C3R: complement receptor 3
C4: complement component 4
C4R: complement receptor 4
C5a: complement component 5a
C5aR: C5a receptor
C5b: complement component 5b
C5b-9: membrane attack complex (MAC)
CCL2: C-C motif chemokine ligand 2
CCL3: C-C motif chemokine ligand 3
CD80: cluster of differentiation 80
CD86: cluster of differentiation 86
CNS: central nervous system
CSF: cerebrospinal fluid
CX3CL1: C-X3-C motif chemokine ligand 1 (fractalkine)
CX3CR1: C-X3-C motif chemokine receptor 1
CXCL13: C-X-C motif chemokine ligand 13
CXCL8: C-X-C motif chemokine ligand 8 (interleukin-8)
CXCL9: C-X-C motif chemokine ligand 9
CXCR5: C-X-C chemokine receptor type 5
DAMPs: damage-associated molecular patterns
DAP12: DNAX-activation protein 12
EAE: experimental autoimmune encephalomyelitis
Fc*γ*R: Fc gamma receptors
GM-CSF: granulocyte macrophage colony-stimulating factor
HATs: histone acetyltransferases
HDACs: histone deacetylases
ICI: immune checkpoint inhibitor
ICOS: inducible T-cell costimulator
IFN: interferon
IGF-1: insulin-like growth factor 1
IL: interleukin
IL-6R: interleukin-6 receptor
irAEs: immune-related adverse events
ITIM: immunoreceptor tyrosine-based inhibitory motif
ITK: interleukin-2-inducible T-cell kinase
ITSM: immunoreceptor tyrosine-based switch motif
MAPK: mitogen-activated protein kinase
MHC II: major histocompatibility complex class II
miR-124: microRNA-124
miR-146a: microRNA-146a
miR-155: microRNA-155
MMP-9: matrix metalloproteinase-9
MS: multiple sclerosis
mTOR: mechanistic target of rapamycin
NF-κB: nuclear factor kappa-light-chain-enhancer of activated B cells
NMOSD: neuromyelitis optica spectrum disorder
PAMPs: pathogen-associated molecular patterns
PD-1: programmed cell death protein 1
PD-L1: programmed cell death ligand 1
PI3K: phosphatidylinositol 3-kinase
PPAR-γ: peroxisome proliferator-activated receptor-γ
ROS: reactive oxygen species
SHP-2: Src homology 2 domain-containing protein tyrosine phosphatase-2
SOCS1: suppressor of cytokine signaling 1
sPD-1: soluble programmed cell death protein 1
sPD-L1: soluble programmed cell death ligand 1
STAT: signal transducer and activator of transcription
sTREM2: soluble triggering receptor expressed on myeloid cells 2
TCR: T-cell receptor
TGF: transforming growth factor
Tfh: T follicular helper
Th1: T helper 1
Th17: T helper 17
TNF: tumor necrosis factor
Treg: regulatory T cell
REM2: triggering receptor expressed on myeloid cells 2
